# Novel association of severe neonatal encephalopathy and Hirschsprung disease in a male with a duplication at the Xq28 region

**DOI:** 10.1186/1471-2350-11-137

**Published:** 2010-09-22

**Authors:** Raquel M Fernández, Rocío Núñez-Torres, Antonio González-Meneses, Guillermo Antiñolo, Salud Borrego

**Affiliations:** 1 Unidad de Gestión Clínica de Genética, Reproducción y Medicina Fetal, Instituto de Biomedicina de Sevilla (IBIS), Hospital Universitario Virgen del Rocío/CSIC/Universidad de Sevilla, Sevilla, Spain; 2 Centro de Investigación Biomédica en Red de Enfermedades Raras (CIBERER), Sevilla, Spain; 3 Unidad de Gestión Clínica de Pediatría, Hospital Universitario Virgen del Rocío, Avda Manuel Siurot s/n, 41013, Sevilla, Spain

## Abstract

**Background:**

Hirschsprung disease (HSCR) is a neurocristopathy characterized by the absence of parasympathetic intrinsic ganglion cells in the submucosal and myenteric plexuses along a variable portion of the intestinal tract. In approximately 18% of the cases HSCR also presents with multiple congenital anomalies including recognized syndromes.

**Methods:**

A combination of MLPA and microarray data analysis have been undertaken to refine a duplication at the Xq28 region.

**Results:**

In this study we present a new clinical association of severe neonatal encephalopathy (Lubs syndrome) and HSCR, in a male patient carrying a duplication at the Xq28 region which encompasses the *MECP2 *and *L1CAM *genes.

**Conclusions:**

While the encephalopathy has been traditionally attributed to the *MECP2 *gene duplication in patients with Lubs syndrome, here we propose that the enteric phenotype in our patient might be due to the dosage variation of the L1CAM protein, together with additional molecular events not identified yet. This would be in agreement with the hypothesis previously forwarded that mutations in *L1CAM *may be involved in HSCR development in association with a predisposing genetic background.

## Background

Hirschsprung disease (HSCR, OMIM 142623) is a congenital malformation of the hindgut characterised by the absence of parasympathetic intrinsic ganglion cells in the submucosal and myenteric plexuses. It is regarded as the consequence of the premature arrest of the craniocaudal migration of vagal neural crest cells in the hindgut between the fifth and 12^th ^week of gestation to form the enteric nervous system (ENS) and is therefore regarded as a neurocristopathy [1]. HSCR occurs as an isolated trait in 70% of cases. Genetic mapping in families and mutational screening of candidate genes, together with the study of several natural and knockout animal models, clearly have shown the involvement of multiple genes in the pathogenesis of HSCR [1,2]. Of them, *RET *proto-oncogene is the major gene involved in the disease, with classical coding hypomorphic mutations accounting for 50% of familial cases and 7-35% of sporadic patients [2], and a common variant within a transcriptional enhancer of its intron 1 which seems to have a prominent role for a great proportion of sporadic cases [3].

On the other hand, a chromosomal abnormality is associated in 12% of HSCR cases, trisomy 21 being by far the most frequent (> 90%). Associated congenital anomalies are found in 18% of the HSCR patients. Among those HSCR-associated syndromes, there exist some clinical presentations with central nervous system anomalies, including the HSAS/MASA spectrum (OMIM 307000 and 303350) ascribed to mutations in the X-linked *L1CAM *gene [1,4]. Indeed, until now mutations of the *L1CAM *gene (OMIM 308840) have been found in nine out of ten patients reported to show association of X-linked hydrocephalus with HSCR [4-10]. In addition, two cases with both acrocallosal syndrome and HSCR have been also linked to *L1CAM *mutations [11]. Therefore, although the question of *L1CAM *being a modifier gene in HSCR has been raised with no definitive answer given thus far [7,8], the whole data suggest that mutations in *L1CAM *may be involved in HSCR development in association with a predisposing background [9].

In this report we present a clinical case of neonatal severe encephalopathy (OMIM 300673) associated with HSCR, presenting with a duplication in the X chromosome encompassing the *MECP2 *(OMIM 300005) and *L1CAM *genes among others. To our knowledge this is the first time in which this kind of association has been described and an extensive analysis has been performed in order to completely dissect its molecular cause.

## Methods

### Patients

The affected patient was the second child born to a healthy couple. He was born at full term with a birth weight of 3.500 Kg. He was hypotonic and failed to thrive in the neonatal period. The patient presented with severe constipation, a distended abdomen, and intestinal biopsy confirmed Hirschsprung disease with aganglionosis extending up to the sigmoid. During his first years of life he developed tonic-clonic seizures and frequent respiratory tract infections. MRI scan showed a discrete increase of the subarachnoid space and dilatation of the ventricular system, cavum septum pellucidum and cavum vergae. He was clinically diagnosed with neonatal encephalopathy, benign external hydrocephalus and sideropenia. The child is currently 13 years old and has not developed speech nor walked, although he can sit without support. He is dysmorphic with a long thin face, down slanting palpebral fissures, divergent strabismus, prognatism, marked ojival palate, long fingers and bilateral cryptorchid. He also presents bilateral hip dislocation. Molecular testing for Angelman and Rett syndrome was negative, and karyotype was normal.

**Figure 1:**
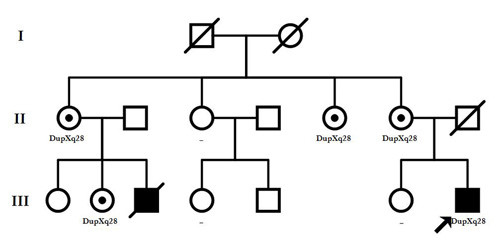
**Pedigree of the patient carrying the duplication at Xq28**. Together with the index patient (III.7), individuals II.1, II.5, II.6 and III.2 were found to be healthy carriers of the rearrangement, while II.3, III.4 and III.6 resulted negative for the testing.

As shown in the family tree (Figure 1), our index patient had a cousin who shared some of his clinical signs and died at the age of 1, fifteen years ago. Clinical reports for this patient describe a congenital midline malformation, with unspecified ventricular alterations and partial agenesis of the corpus callosum. Unfortunately no additional clinical information is available for this patient.

We had also available the DNA from the mother, sister, three maternal aunts and two maternal cousins of our index patient (individuals II.1, II.3, II.5, II.6, III.2, III.4 and III.6 of the family tree). An informed consent was obtained from all the participants for clinical and molecular genetic studies. The study conformed to the tenets of the declaration of Helsinki and was approved by the "Committee of Ethics and Clinical Investigation" from Hospitales Universitarios Virgen del Rocío.

### MLPA analysis

The MLPA (Multiple Ligation Probe-dependent Amplification) method employed was as described by Schouten et al. [12], using MLPA kits purchased from MRC-Holland (Amsterdam, The Netherlands), and according to manufacturer's recommendations. MLPA is a variation of the PCR that permits multiple targets to be amplified with only a single primer pair. Each probe consists of a two oligonucleotides which recognise adjacent target sites on the DNA. One probe oligonucleotide contains the sequence recognized by the forward primer, the other the sequence recognised by the reverse primer. Only when both probe oligonucleotides are hybridized to their respective targets, can they be ligated into a complete probe. Each complete probe has a unique length, so that its resulting amplicons can be separated and identified by capillary electrophoresis. Since the forward primer used for probe amplification is fluorescently labeled, each amplicon generates a fluorescent peak which can be detected by a capillary sequencer. Comparing the peak pattern obtained on a given sample with that obtained on various reference samples, the relative quantity of each amplicon can be determined. Initial MLPA studies were aimed to completely rule out the possibility of any microdeletion syndrome included in the P245 kit. When a duplication was observed for the three probes binding the *MECP2 *gene, MLPA was performed using the P015D kit, which included probes for the four exons of this gene, together with *IRAK1*, *L1CAM*, *IDH3G *and *SLC6A8*, alongside 12 X chromosome and four autosomal control probes. The P106 kit for X-linked mental retardation syndromes, comprising 3 genes at Xq28 (*GDI1, AFF2, SLC6A8*) and several other genes along the whole X chromosome was also employed to analyze the extent of the duplication. Subsequently and in order to verify the results, we used the P049 kit which covered a series of genes at Xq.28 as *FMR2, IDS, PNCK, SLC6A8, BCAP31, ABCD1, IDH3G, L1CAM, IRAK1, MECP2, FLNA, GDI1, FVIII *and *SYBL1*, and also included six chromosome X control probes. Fragment analyses were performed using the 3730 DNA analyzer (Applied Biosystems, Foster City, CA, USA) and for data analysis we used GeneMarker v 1.6 (Softgenetics L.L.C). We normalized the samples by the peak height, and included control individuals who had previously been confirmed to have no CNVs of the studied genes. Furthermore, the assays were performed in duplicated to confirm the robustness of the analysis.

### Microarray analysis

DNA samples from our patient and his mother were hybridized with Genome wide human SNP 6.0 arrays (Affymetrix), in order to analyze the presence CNVs. Samples (20 μl) diluted to 50 ng/μl were processed in the GeneChip^® ^Instrument System platform (Affymetrix UK L) following the manufacturer's recommendations. Copy number data were generated by comparing intensities for both SNP and copy number probes ''in silico'' to the HapMap control provided by Affymetrix. The resulting log2ratios were then analysed using a Hidden Markov Model (HMM) to generate copy number calls for each probe. The quality of the log2 data was assessed by the degree of variation, determined by the MAPD metric. MAPD is defined as the Median of the Absolute value of all Pairwise Differences between log2 ratios for a given chip. High MAPD > 0.4 (using the HapMap control) is considered to be the cut-off at which copy numbers can no longer be accurately called.

### X inactivation analysis

X inactivation assays performed for the female subjects of the family as well as for controls, consisted in the examination of the human androgen-receptor gene (*HUMARA*, Xq11.2) methylation status, as described by Allen et al. [13]. This study was pertinent to explain the complete absence of any clinical manifestation in all the female carriers of the rearrangement within this family. Samples were separated on an ABI3730 automated DNA sequencer (Applied Biosystems) and were analyzed with GeneMapper software for peak positions and area intensity calculations. These data were further processed with the use of Excel.

### Mutational screening, SNPs genotyping and haplotyping

PCR - dHPLC analysis was undertaken for the mutational screening of *RET *and other HSCR related genes such as *EDNRB, EDN3, SOX10, PHOX2B, GDNF and NTN*, using previously described conditions [14,15]. The whole coding sequence of the *L1CAM *gene was also screened in both the affected child and his mother by direct sequencing (conditions available on request).

*RET *SNPs c.73-126G > T (rs2565206), c.73-1370C > T (rs2505532), c.73-1463T > C (rs2505533) and c.73+9277T > C (rs2435357) were genotyped in our patient employing Taqman based techniques for allelic discrimination (TaqMan^® ^SNP Genotyping Assay, Applied Biosystems, Foster City, CA) as previously described [16].

## Results

**Figure 2:**
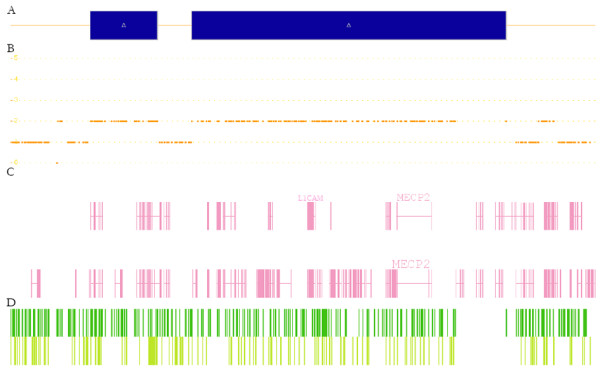
**Representation of the duplicated region in our patient refined with the Genome wide human SNP 6.0 arrays (Affymetrix)**.

**Figure 3:**
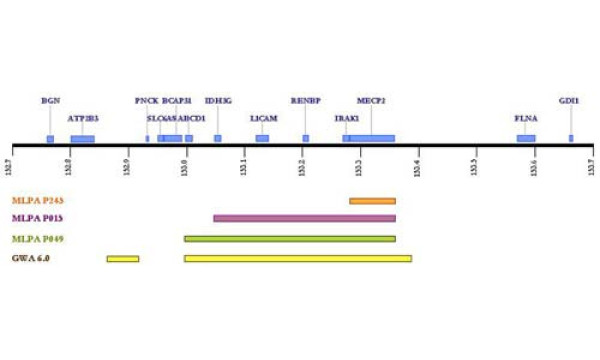
**Schematic representation of the genomic region at Xq28 involved in the duplication in our patient**. The regions covered by each of the MLPA sets of probes (P245, P015 and P049) are indicated. The refinement of the region using the Genome wide human SNP 6.0 array of Affymetrix is also shown.

Investigation of the affected patient using the different MLPA kits demonstrated a duplication at Xq28 including *MECP2, IRAK1, L1CAM, IDH3G *and a region of *ABCD1 *from exon 3 upstream. Normal dosages were observed for exons 1 and 2 of *ABCD1 *as well as for the remaining neighbouring genes *GDI1*, *SLC6A8, BCAP31 *or *PNCK*. Taking into account the relative positions of all these genes, the duplication seemed to comprise approximately a region of a minimum of 297 Kb. Subsequently, by performing copy number analysis using the Affymetrix 6.0 SNP chip, we were able to confirm the presence of the rearrangement and characterized it in detail (Figure 2). As shown in figure 3, a duplication encompassing 621 Kb and containing two stretches of 65,8 Kb and 35.1 Kb of non-duplicated sequences, was detected in our index patient.

Segregation analysis of the family by MLPA showed that the duplication was not a *de novo *event but had been inherited from his mother, and also was carried by other four healthy females within the family, including the mother of the deceased cousin (Figure 1). X inactivation testing on the DNA samples for all the asymptomatic female carriers, showed a marked skewed inactivation with the inactive X chromosome being the duplicated one (99,999975% of active normal allele versus 0,000025% of active mutant allele), which would clearly explain the absence of any clinical feature in those individuals.

On the other hand, the mutational screening of the coding regions of *RET *and the remaining genes tested including *L1CAM*, revealed no mutations which could be attributable to HSCR phenotype in our patient. Moreover molecular testing of the *RET *haplotype comprising specific variants within its intron 1, showed that this patient did not carry the "HSCR-associated combination" [17,18], nor the so-called "enhancer mutation" [3].

## Discussion

Over 50 patients have to date been reported with Xq28 duplications varying in size from 0.2 Mb to larger scale duplications extending beyond Xq27. Most have included the *MECP2 *and *L1CAM *genes, although none of them have been reported to present with Hirschsprung disease [19-21]. It has been suggested that the clinical features associated to neonatal encephalopathy in males carrying the Xq28 duplication might be solely due to the *MECP2 *gene, as patients with duplications involving this region alone have been reported with the same classical phenotype [19,21]. Indeed, an OMIM number is used to specifically design the disorder caused by duplication or triplication of *MECP2 *(OMIM#300260; Lubs X-Linked mental retardation syndrome). Although spasticity, severe learning disability, axial hypotonia and frequent chest infections have been common findings within this group of patients, gastrointestinal symptoms have been much rarer. Only worth of note, in the report by Clayton-Smith et al., eight families with several male affected patients carrying Xq28 duplications had presented with intestinal pseudo-obstruction or bladder distension, but these features were attributed exclusively to the duplication of the *FLNA *gene [21]. Several arguments supported such assumption, since these authors had also found 2 other families carrying intragenic duplications of *FLNA *and presenting exclusively the enteric phenotype. Moreover, Gargiulo et al. had already reported point *FLNA *mutations in X-linked families with pseudo-obstruction [22]. The pathogenic mechanism proposed by the authors to explain how both duplications and truncating mutations of the *FLNA *gene could lead to intestinal pseudo-obstruction, was the hypothesis that the amount of FLNA would be critical for neuronal migration, and either its increased or decreased expression could interfere with the normal migration process [21]. In our particular case we could propose exactly the same pathogenic mechanism for the connection between the *L1CAM *gene and Hirschsprung disease in our patient carrying the duplication at Xq28. The L1 cell adhesion molecule is a membrane glycoprotein belonging to a large class of immunoglobulin superfamily cell adhesion molecules (CAMs) that mediate cell-to-cell adhesion at the cell surface. The L1CAM protein is found primarily in the nervous system and is important in neuronal adhesion, migration, neurite outgrowth, and myelination [4]. Mutations in the *L1CAM *gene cause neurological abnormalities of variable severity, including congenital hydrocephalus, agenesis of the corpus callosum, spastic paraplegia, bilaterally adducted thumbs, aphasia, and mental retardation (see OMIM). Interestingly, *L1CAM *is the only gene included in the duplicated region which has been verified to be a ENS-expressed gene and to play a key role in its development during embryogenesis [23], and some cases of patients with either X-linked hydrocephalus or acrocallosal syndrome and HSCR have been reported to present *L1CAM *mutations (visit HGMD http://www.hgmd.cf.ac.uk/ac/all.php). In contrast, none of the remaining genes encompassed by the rearrangement, such as *IRAK1 *or *IDH3G *have been previously related to HSCR. All the *L1CAM *mutational events reported so far to be responsible for the manifestation of the corresponding clinical pictures, are point sequence changes [4,6,8,10,11] or small deletions [5,9], whose proposed pathogenic mechanisms lead to the suspicion that decreased L1CAM may be a modifying factor in the development of HSCR [4]. It would be licit to speculate that also an increase in the amount of L1CAM, due to a duplication in the dosage of this gene, might be involved someway in the pathogenesis of Hirschsprung in this particular patient. In other words it would be plausible that the amount of L1CAM would be critical during ENS development, and either an increase or decrease of this protein could interfere with the processes of neuronal adhesion and migration.

The association between encephalopathy and HSCR should not be considered surprising as brain development is largely controlled by the same neural growth factors acting in the ENS [24]. Because the frequency of Lubs X-linked mental retardation syndrome is estimated to be extremely low (only around 50 cases being reported to date), and the frequency of HSCR is approximately 1:5000, then the anticipated incidence of both together would be almost inexistent by chance alone. So it is not probable that the association of both clinical phenotypes has occurred accidentally and by different molecular events. This statement is supported by the fact that L1CAM abnormality has been previously proposed to contribute to HSCR onset [8,9,25]. Parisi et al. hypothesized that in those cases in which X-linked hydrocephalus presents together with HSCR, either *RET *or another HSCR gene contributes to aganglionosis under the influence of a defective *L1CAM *gene, and *L1CAM *may act as an X linked modifier gene for the development of HSCR [8]. We therefore propose a similar role for *L1CAM *in the context of the association of Lubs syndrome and HSCR. Given the complex nature of HSCR, it would be conceivable that the duplication at Xq28 region including the *L1CAM *gene, together with another still unidentified molecular events, could lead to the manifestation of the disease in our patient. The existence of those additional molecular events would also explain why no other patients carrying MECP2-L1CAM duplications reported to date present with HSCR [19-21]. In addition, we have also to argue that children with congenital severe neonatal encephalopathy cannot communicate symptoms such as constipation, and originally, they have a constipation tendency; so there may be several cases of HSCR among those patients that goes undiagnosed because of a premature death, just as it occurred with the cousin of our index patient.

## Conclusions

Although a more comprehensive analysis of additional HSCR genes is warranted, our results support an involvement of *L1CAM *duplication in the pathogenesis of HSCR, and present evidence of a possible novel syndromic form of Hirschsprung disease.

## Competing interests

The authors declare that they have no competing interests.

## Authors' contributions

RMF and RN-T carried out the molecular genetic studies. AG-M delineated the clinical presentation of the index patient and recruited the family. SB coordinated and supervised all the analyses. RMF and SB drafted the manuscript, and GA collaborated with valuable contributions to the manuscript. All authors have read and approved the final manuscript.

## Pre-publication history

The pre-publication history for this paper can be accessed here:

http://www.biomedcentral.com/1471-2350/11/137/prepub
